# Hypoxia induces food leaving in *C. elegans*

**DOI:** 10.17912/micropub.biology.000776

**Published:** 2023-03-22

**Authors:** Longjun Pu, Lina Zhao, Qiongxuan Lu, Changchun Chen

**Affiliations:** 1 Umeå Centre for Molecular Medicine, Umeå University, Umeå, Sweden; 2 Department of Molecular Biology, Umeå University, Umeå, Sweden; 3 Wallenberg Centre for Molecular Medicine, Umeå University, Umeå, Sweden

## Abstract

Hypoxia alters eating behavior in different animals. In *C. elegans* , hypoxia induces a strong food leaving response. We found that this behavior was independent of the known O _2_ response mechanisms including acute O _2_ sensation and HIF-1 signaling of chronic hypoxia response. Mutating *egl-3* and *egl-21* , encoding the neuropeptide pro-protein convertase and carboxypeptidase, led to defects in hypoxia induced food leaving, suggesting that neuropeptidergic signaling was required for this response. However, we failed to identify any neuropeptide mutants that were severely defective in hypoxia induced food leaving, suggesting that multiple neuropeptides act redundantly to modulate this behavior.

**Figure 1. Hypoxia evokes food leaving response in C. elegans:**
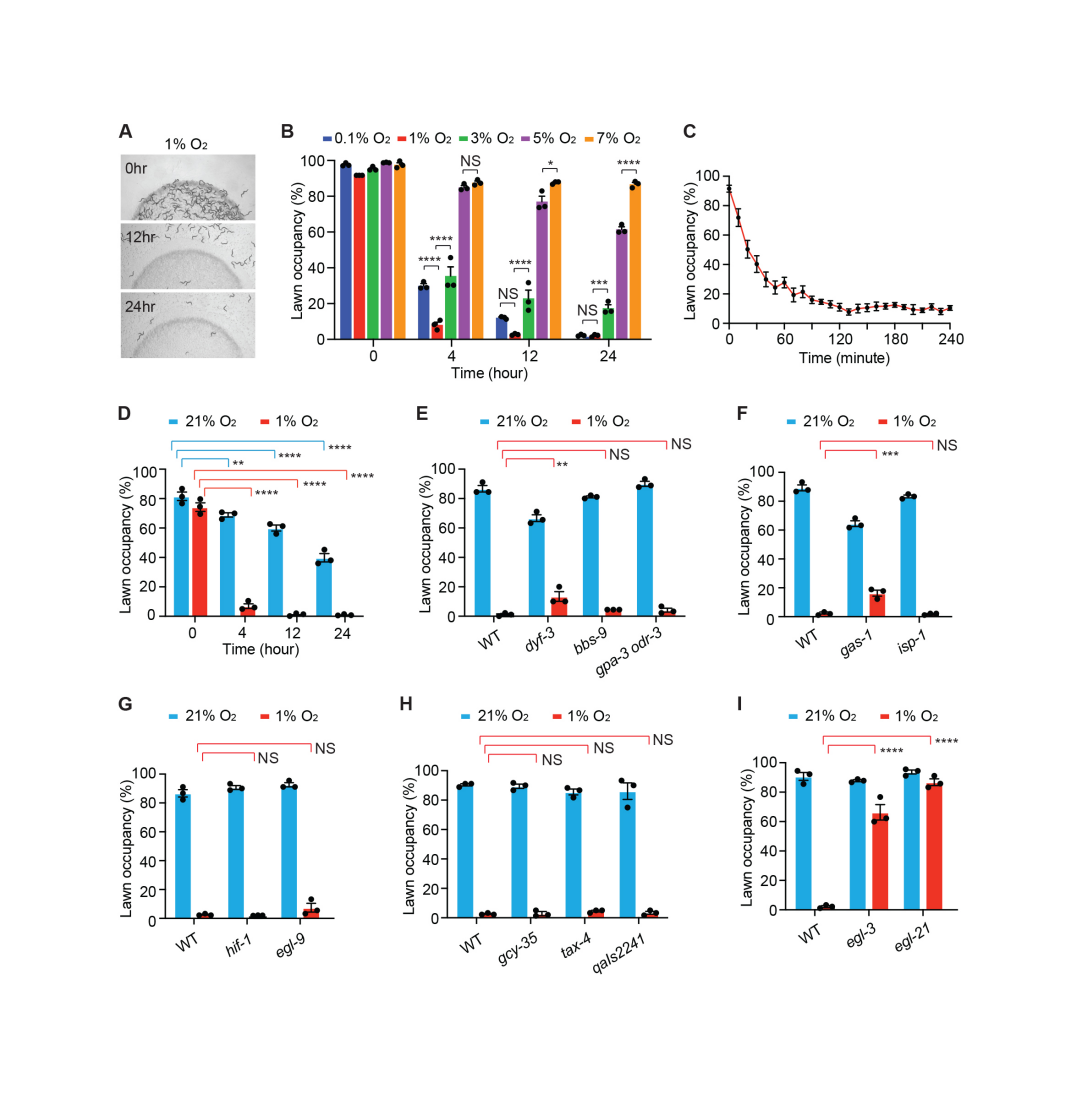
**(A)** Representative images showing food leaving of wild type animals ( N2 ) in 1% O _2_ for 0, 12 and 24 hours. **(B)** Lawn occupancy of wild type animals in the indicated O _2_ concentrations for 0, 4, 12 and 24 hours. Error bars indicate standard error of the mean (SEM). Three independent experiments were performed with three technical replicates for each strain in each experiment. *, *p* <0.05; ***, *p* <0.001; ****, *p* <0.0001; NS = not significant. One way ANOVA with Tukey's test. **(C)** Video tracking of lawn occupancy of wild type animals in 1% O _2_ for 4 hours. 5 videos were acquired. The average lawn occupancy of 5 videos was plotted every 10 minutes. Error bars indicate SEM. **(D)** Lawn occupancy of wild type animals on dead bacteria for 0, 4, 12 and 24 hours in 21% O _2_ (blue) and 1% O _2_ (red). Three independent experiments were performed with three technical replicates for each strain in each experiment. **, *p* <0.01; ****, *p* <0.0001. One way ANOVA with Tukey's test. **(E)** Lawn occupancy of wild type, *dyf-3 ( m185 )* , *bbs-9 ( gk471 )* and *gpa-3 ( pk35 ) odr-3 ( n1605 )* in 21% O _2_ (blue) and 1% O _2_ (red) for 24 hours. Three independent experiments were performed with three technical replicates for each strain in each experiment. **, *p* <0.01; NS = not significant. One way ANOVA with Tukey's test. **(F)** Lawn occupancy of wild type, *gas-1 ( fc21 )* and *isp-1 ( qm150 )* mutants in 21% O _2_ (blue) or 1% O _2_ (red) for 24 hours. Three independent experiments were performed with three technical replicates for each strain in each experiment. ***, *p* <0.001; NS = not significant. One way ANOVA with Tukey's test. **(G)** Lawn occupancy of wild type, *hif-1 ( ia4 )* and *egl-9 ( sa307 )* in 21% O _2_ (blue) and 1% O _2_ (red) for 24 hours. Three independent experiments were performed with three technical replicates for each strain in each experiment. NS = not significant. One way ANOVA with Tukey's test. **(H)** Lawn occupancy of wild type *gcy-35 ( ok769 )* , *tax-4 ( p678 )* and *qaIs2241* in 21% O _2_ (blue) or 1% O _2_ (red) for 24 hours. In the *qaIs2241* strain, the O _2_ sensing neuron URX, AQR and PQR were genetically ablated. Three independent experiments were performed with three technical replicates for each strain in each experiment. NS = not significant. One way ANOVA with Tukey's test. **(I)** Lawn occupancy of wild type, *egl-3 ( ok979 )* and *egl-21 ( n476 )* in 21% O _2_ (blue) or 1% O _2_ (red) for 24 hours. Three independent experiments were performed with three technical replicates for each strain in each experiment. ****, *p* <0.0001. One way ANOVA with Tukey's test.

## Description

The exposure to hypoxia at high altitude often triggers the inhibition of appetite and causes the loss of body weight (Kayser & Verges, 2021; Kietzmann & Makela, 2021; Quintero, Milagro, Campion, & Martinez, 2010; Westerterp, Kayser, Wouters, Le Trong, & Richalet, 1994). Hypoxia induced anorexia has also been observed in the nematode *C. elegans* (Figure 1A) (Abergel, Shaked, Shukla, Wu, & Gross, 2021; Gross et al., 2014; Van Voorhies & Ward, 2000). The neuroglobin GLB-5 and the phosphatidylinositol transfer protein PITP-1 have been reported to regulate the recovery from hypoxia evoked food leaving (Abergel et al., 2021; Gross et al., 2014). In this study, we aimed to identify the molecules, which were required for animals to leave the bacterial lawn under hypoxia (Figure 1A). *C. elegans* displayed an O _2_ dependent escaping from the food lawn. The majority of animals left the food after 4 hours in 3% O _2_ , whereas 5% O _2_ did not effectively trigger food leaving response even though there was a significant reduction of lawn occupancy after 12 or 24 hours in 5% O _2_ (Figure 1B). The strongest food leaving response was observed at 1% O _2_ (Figure 1B). Intriguingly, the response was less robust at 0.1% O _2_ (Figure 1B). Therefore, 1% O _2_ was used for the rest of our analysis. We next continuously tracked the food leaving events in 1% O _2_ for 4 hours. Animals quickly escaped from the bacterial lawn once they were exposed to 1% O _2_ , and nearly all the animals left the food after 2 hours in 1% O _2_ (Figure 1C). When animals encountered the bacteria again, they immediately initiated reversals and remained outside the lawn (Extended Data), suggesting that they avoid food under hypoxia.

One plausible mechanism underlying this phenomenon was that bacterial metabolites generated under hypoxia might be aversive to *C. elegans* . Therefore, we assayed hypoxia induced food leaving using heat-killed bacteria. *C. elegans* robustly left the dead bacteria at 1% O _2_ (Figure 1D), which excluded this possibility. We also noticed that a significant proportion of animals left the dead bacteria after 12 and 24 hours at 21% O _2_ (Figure 1D), probably due to poor food qualities. We next sought to probe the potential mechanisms of hypoxia evoked food leaving, and began with the examination if the known O _2_ response machineries were involved. *C. elegans* markedly increased its locomotory speed when O _2_ level rapidly dropped to hypoxia (Onukwufor et al., 2022; Zhao et al., 2022). *dyf-3* , *bbs-9* , and *gpa-3 odr-3* mutants, which were defective in speed response to hypoxia (Zhao et al., 2022), appeared to exhibit strong food leaving in 1% O _2_ (Figure 1E). Similarly, the mitochondrial mutants, *gas-1* and *isp-1* that were unable to increase their locomotory speed in hypoxia (Zhao et al., 2022), also exhibited robust escaping from the food in 1% O _2_ (Figure 1F), with significant but mild defects observed in *gas-1* and *dyf-3* mutants (Figure 1E and F). These observations suggest distinct mechanisms underlie hypoxia induced food leaving and acute speed response to hypoxia. We subsequently explored if chronic hypoxia response contributed to hypoxia induced food leaving. With sufficient O _2_ supply, the proline-4-hydroxylase PHD/ EGL-9 modifies HIF-1 for the degradation, a process that is mediated by the von Hippel-Lindau (VHL) tumor suppressor protein (Kaelin & Ratcliffe, 2008; Semenza, 2010). The disruption of PHD/ EGL-9 or VHL stabilizes HIF-1 . We found that disrupting *hif-1* or *egl-9* did not affect the hypoxia induced escaping from bacterial lawn (Figure 1G), suggesting that HIF-1 signaling is not required for this response. The other well described O _2_ response mechanism in *C. elegans* is the sensation of 21% O _2_ . This is mediated by the O _2_ sensing neurons URX, AQR and PQR, and requires soluble guanylate cyclase GCY-35 and cGMP gated channel TAX-4 (Busch et al., 2012; Cheung, Cohen, Rogers, Albayram, & de Bono, 2005; Couto, Oda, Nikolaev, Soltesz, & de Bono, 2013; Gray et al., 2004; Laurent et al., 2015; Zimmer et al., 2009). However, *gcy-35* and *tax-4* mutants as well as *qaIs2241* strain that lacked O _2_ sensing neurons displayed robust food leaving under hypoxia (Figure 1H). Taken together, these observations suggest that animals leave the bacterial food under hypoxia via a distinct O _2_ response mechanism.

To gain further insight into hypoxia induced anorexia in *C. elegans* , we performed a candidate screen for mutants that were defective in this response (Table 1 in Reagents). We discovered that *egl-3* and *egl-21* mutants were defective in hypoxia induced food leaving (Table 1 in Reagents). Over 60% of both *egl-3* and *egl-21* mutants remained on the bacterial lawn after 24 hours in 1% O _2_ (Figure 1I). *egl-3* encodes a pro-peptide convertase required for neuropeptide precursor cleavage (Kass, Jacob, Kim, & Kaplan, 2001), and *egl-21* encodes a carboxypeptidase that removes C-terminal basic residues from peptide sequences (Jacob & Kaplan, 2003), suggesting that neuropeptidergic signaling is required for hypoxia induced food avoidance. These observations prompted us to probe which neuropeptide was involved. We assayed 36 strains, in which all neuropeptide genes were disrupted, for their food leaving behavior under hypoxia (Table 1 in Reagents). Surprisingly, we did not recover any strains that were as defective as *egl* mutants (Table 1 in Reagents), suggesting that multiple neuropeptides might act redundantly to regulate the food leaving under hypoxia.

## Methods

***C. elegans* maintenance**

*C. elegans***** strains were maintained under standard conditions (Brenner, 1974). The Bristol N2 were used as wild type. Strains used in this study were listed in Table 1.

**Behavioral analysis under hypoxia**

To analyze hypoxia induced food leaving events, three biological replicates were performed for each strain, and 3 technical replicates were included in each biological replicate. The assay was performed as the following. 5.5 cm assay plates were seeded with a 1 cm diameter OP50 lawn two days before use. 100 day-one adults were picked to each assay plate and were allowed to settle down for 30 minutes in the room air. The number of animals remained on the bacterial lawn after 30 minutes in 21% O _2_ was counted and used as the lawn occupancy of time 0. The assay plates were subsequently exposed to the defined O _2_ concentrations (0.1%, 1%, 3%, 5% or 7% O _2_ balanced with nitrogen) in the hypoxia chamber (Don Whitley M85 workstation). The temperature in the chamber was set to 21°C, which was close to the room temperature in the lab. A Zeiss Stemi 508 microscope coupled with a Grasshopper camera (FLIR) and a laptop was placed in the hypoxia chamber to capture images and videos. Images were collected after assay plates were transferred to the chamber for 4, 12 and 24 hours. The number of animals on the food lawn could be easily counted in the high-resolution images. The assays were also run in parallel at 21% O _2_ , and the lawn occupancy at 21% O _2_ at each time point was used as the control. To monitor the dynamic food leaving events, videos were recorded with 2 frames per second for 4 hours. The recording was started immediately when assay plates were placed into the hypoxia chamber. 5 videos of 4 hours were collected at 5 different days. The number of animals on the bacteria lawn were counted every 10 minutes for each video.

To assay hypoxia induced escaping from the dead bacteria, 100 ml of OP50 was grown overnight at 37°C followed by the incubation at 70°C for 4 hours, which efficiently killed the bacteria. The dead bacteria were concentrated, and seeded on the assay plates to generate a 1 cm bacterial lawn. The assay was performed as described above.

In the candidate screen, 30 day-one adult animals were picked to each assay plate, and each strain was assayed 3 times, with one plate per day at 3 different days. The data in Table 1 were the average lawn occupancy of 3 assays.

**CRISPR/Cas9 genome editing**

The *yum* alleles were all generated using CRISPR/Cas9 mediated genome editing as outlined previously (Dokshin, Ghanta, Piscopo, & Mello, 2018; Ghanta & Mello, 2020). The strategy involved the homology-directed integration of single strand DNA oligo (ssODN). The ssODN templates contained two homology arms of 35 bases flanking the targeted PAM sites. The insertion of ssODN introduced one in-frame and two out-of-frame stop codons as well as a unique restriction enzyme cutting site for genotyping. 16bp coding sequence was simultaneously deleted to generate frameshift. The ribonucleoprotein complexes were assembled by mixing 0.5μl of Cas9 protein (IDT), 5μl of 0.4μg/μl tracrRNA (IDT), and 2.8μl of 0.4μg/μl crRNA (IDT), and incubated at 37°C for 10 minutes. 2.2μl of 1μg/μl ssODN, 2μl of 600μg/μl *rol-6* co-injection, and 7.5μl nuclease free water were subsequently added to the final volume of 20μl. The mixture was centrifuged at maximum speed for 5 minutes and loaded into micro-capillary for injection. The F1 roller animals were picked and genotyped for the integration of ssODN. The detailed sequence information of crRNA, ssODN and genotyping primers are available upon request.

## Reagents

**Table:** 

	**Strain**	**Genotype**	**Source**	**Lawn occupancy** ± **SEM (%), 24 hours in 1% O _2_**
	N2	Wild type	CGC	1.11 ± 1.1%
**Ciliogenesis**	SP1603	*dyf-3 ( m185 ) IV. *	CGC	10.00 ± 1.9%
	VC1062	*bbs-9 ( gk471 ) I. *	CGC	4.44 ± 2.9%
	PR802	*osm-3 ( p802 ) IV. *	CGC	1.11 ± 1.1%
	VC1228	*klp-11 ( tm324 ) IV. *	CGC	1.11 ± 1.1%
	CB3330	*che-11 ( e1810 ) V. *	CGC	0.00 ± 0%
	CB1124	*che-3 ( e1124 ) I. *	CGC	0.00 ± 0%
	MX124	*ifta-1 ( nx61 ) X. *	CGC	0.00 ± 0%
**Biogenic amine related**	MT15434	*tph-1 ( mg280 ) II. *	CGC	5.56 ± 2.9%
	LC33	*bas-1 ( tm351 ) III. *	CGC	4.44 ± 2.9%
	BZ873	*dop-3 ( ok925 ) X. *	CGC	3.33 ± 1.9%
	MT9455	*tbh-1 ( n3247 ) X. *	CGC	2.22 ± 1.1%
	CB1112	*cat-2 ( e1112 ) II. *	CGC	1.11 ± 1.1%
	LC81	*cat-4 ( tm773 ) V. *	CGC	0.00 ± 0%
	LX636	*dop-1 ( vs101 ) X. *	CGC	0.00 ± 0%
	FG58	*dop-4 ( tm1392 ) X. *	CGC	0.00 ± 0%
**Channels**	PR678	*tax-4 ( p678 ) III. *	CGC	5.56 ± 2.9%
	VC1233	*ocr-2 ( ok1711 ) IV. *	CGC	4.44 ± 2.2%
	RB1556	*shw-3 ( ok1884 ) V. *	CGC	4.44 ± 4.4%
	VC542	*kcc-1 ( ok692 ) IV. *	CGC	3.33 ± 1.9%
	AX1964	*cng-1 (db111) V. *	This study	1.11 ± 1.1%
	RB1374	*ocr-3 ( ok1559 ) X. *	CGC	1.11 ± 1.1%
	CB1126	*che-6 ( e1126 ) IV. *	CGC	1.11 ± 1.1%
	CHS504	*cng-3 ( jh113 ) IV. *	CGC	0.00 ± 0%
	RB753	*lov-1 ( ok522 ) II. *	CGC	0.00 ± 0%
	VC602	*trp-2 ( gk298 ) III. *	CGC	0.00 ± 0%
	EJ1158	*gon-2 ( q388 ) I. *	CGC	0.00 ± 0%
	VC244	*gtl-1 ( ok375 ) IV. *	CGC	0.00 ± 0%
	NM1968	*slo-1 ( js379 ) V. *	CGC	0.00 ± 0%
	LY100	*slo-2 ( nf100 ) X. *	CGC	0.00 ± 0%
	VC1149	*kqt-1 ( ok413 ) X. *	CGC	0.00 ± 0%
	RB883	*kqt-2 ( ok732 ) X. *	CGC	0.00 ± 0%
	VC160	*trp-1 ( ok323 ) III. *	CGC	0.00 ± 0%
	VC1141	*trp-4 ( ok1605 ) I. *	CGC	0.00 ± 0%
**Guanylate cyclases**	CHS11290	*gcy-11 ( tm8150 ) X. *	NBRP, Japan	8.89 ± 5.8%
	CHS11291	*gcy-21 (tm11147) II. *	NBRP, Japan	4.44 ± 2.9%
	CHS502	*gcy-28 (yum32) I. *	This study	4.44 ± 2.9%
	OH4844	*gcy-5 ( tm897 ) II. *	CGC	3.33 ± 1.9%
	AX1295	*gcy-35 ( ok769 ) I. *	CGC	2.77 ± 1.4%
	CHS11285	*gcy-1 ( tm2669 ) II. *	NBRP, Japan	2.22 ± 2.2%
	CHS11289	*gcy-9 ( tm7632 ) X. *	NBRP, Japan	2.22 ± 1.1%
	CHS56	*npr-1 ( ad609 ) X; gcy-31 (syb852) X; gcy-32 ( ok995 ) V; gcy-33 (syb842) V; gcy-34 ( ok1012 ) V; gcy-35 ( ok769 ) I; gcy-36 ( db42 ) X; gcy-37 ( ok384 ) IV. *	This study	2.22 ± 1.1%
	VC2796	*gcy-3 ( gk1154 ) II. *	CGC	1.11 ± 1.1%
	CHS11287	*gcy-6 ( tm1449 ) V. *	NBRP, Japan	1.11 ± 1.1%
	CHS11288	*gcy-7 ( tm901 ) V. *	NBRP, Japan	1.11 ± 1.1%
	CX2065	*odr-1 ( n1936 ) X. *	CGC	1.11 ± 1.1%
	RB1935	*gcy-20 ( ok2538 ) V. *	CGC	1.11 ± 1.1%
	CHS11292	*gcy-25 ( tm4300 ) IV. *	NBRP, Japan	1.11 ± 1.1%
	CHS11293	*gcy-27 (tm11852) IV. *	NBRP, Japan	1.11 ± 1.1%
	DR47	*daf-11 (m47) V. *	CGC	1.11 ± 1.1%
	VC3024	*gcy-2 ( ok3721 ) II. *	CGC	0.00 ± 0%
	CHS11286	*gcy-4 ( tm1653 ) II. *	NBRP, Japan	0.00 ± 0%
	IK597	*gcy-23 ( nj37 ) gcy-8 ( oy44 ) gcy-18 ( nj38 ) IV. *	CGC	0.00 ± 0%
	CHS419	*gcy-12 (yum88) II; bbs-9 ( gk471 ) I. *	This study	0.00 ± 0%
	CHS497	*gcy-13 (yum85)V; bbs-9 ( gk471 ) I. *	This study	0.00 ± 0%
	JN1194	*gcy-14 ( pe1102 ) V. *	CGC	0.00 ± 0%
	VC2675	*gcy-15 ( gk1102 ) II. *	CGC	0.00 ± 0%
	VC2450	*gcy-17 ( gk1155 ) I. *	CGC	0.00 ± 0%
	RB1909	*gcy-19 ( ok2472 ) II. *	CGC	0.00 ± 0%
**Globins**	CHS11299	*glb-20 (tm12286) X. *	NBRP, Japan	3.33 ± 1.9%
	CHS521	*glb-27 (yum20) II. *	This study	3.33 ± 1.9%
	CHS534	*glb-32 (yum26) V. *	This study	3.33 ± 1.9%
	FX05440	*glb-5 ( tm5440 ) V. *	NBRP, Japan	2.22 ± 2.2%
	CHS519	*glb-9 (yum19) II. *	This study	2.22 ± 1.1%
	CHS11300	*glb-21 ( tm8033 ) IV. *	NBRP, Japan	2.22 ± 2.2%
	CHS529	*glb-22 (yum24) V. *	This study	2.22 ± 2.2%
	CHS515	*glb-23 (yum17) IV. *	This study	2.22 ± 2.2%
	CHS517	*glb-24 (yum18) V. *	This study	2.22 ± 2.2%
	CHS523	*glb-31 (yum21) II. *	This study	2.22 ± 2.2%
	CHS11303	*glb-33 ( tm3655 ) V. *	NBRP, Japan	2.22 ± 2.2%
	CHS506	*glb-1 (yum12) III. *	This study	1.11 ± 1.1%
	CHS540	*glb-3 (yum29) V. *	This study	1.11 ± 1.1%
	CHS11294	*glb-6 ( tm3795 ) V. *	NBRP, Japan	1.11 ± 1.1%
	CHS541	*glb-8 (yum30) I. *	This study	1.11 ± 1.1%
	CHS509	*glb-11 (yum14) III. *	This study	1.11 ± 1.1%
	CHS543	*glb-12 (yum31) II. *	This study	1.11 ± 1.1%
	CHS11296	*glb-16 ( tm5264 ) X. *	NBRP, Japan	1.11 ± 1.1%
	CHS11302	*glb-28 ( tm6910 ) X. *	NBRP, Japan	1.11 ± 1.1%
	CHS527	*glb-29 (yum23) II. *	This study	1.11 ± 1.1%
	CHS508	*glb-2 (yum13) IV. *	This study	0.00 ± 0%
	CHS535	*glb-7 (yum27) IV. *	This study	0.00 ± 0%
	CHS11295	*glb-10 ( tm5533 ) II. *	NBRP, Japan	0.00 ± 0%
	CHS925	*glb-13 ( tm2825 ) X. *	NBRP, Japan	0.00 ± 0%
	CHS513	*glb-17 (yum16) X. *	NBRP, Japan	0.00 ± 0%
	CHS11297	*glb-18 ( tm6017 ) I. *	NBRP, Japan	0.00 ± 0%
	CHS11298	*glb-19 ( tm6965 ) IV. *	NBRP, Japan	0.00 ± 0%
	CHS531	*glb-25 (yum25) V. *	This study	0.00 ± 0%
	CHS11301	*glb-26 ( tm4837 ) I. *	NBRP, Japan	0.00 ± 0%
	CHS538	*glb-30 (yum28) III. *	This study	0.00 ± 0%
**Mitochondria**	CW152	*gas-1 ( fc21 ) X. *	CGC	18.89 ± 8.6%
	VC3201	*atfs-1 ( gk3094 ) V. *	CGC	3.33 ± 1.9%
	PH13	*rad-8 ( mn163 ) I. *	CGC	2.22 ± 1.1%
	MQ887	*isp-1 ( qm150 ) IV. *	CGC	2.22 ± 1.1%
	MQ130	*clk-1 ( qm30 ) III. *	CGC	1.11 ± 1.1%
	RB2547	*pink-1 ( ok3538 ) II. *	CGC	0.00 ± 0%
	NK2784	*trak-1 (qy158[ trak-1 ::mNG + loxP]) I. *	CGC	0.00 ± 0%
	CZ1998	*mcu-1 ( ju1154 ) IV. *	CGC	0.00 ± 0%
	MQ1333	*nuo-6 ( qm200 ) I. *	CGC	0.00 ± 0%
	TK22	*mev-1 ( kn1 ) III. *	CGC	0.00 ± 0%
	RB2434	*asg-2 ( ok3344 ) X. *	CGC	0.00 ± 0%
**Neuropeptides**	KP2018	*egl-21 ( n476 ) IV. *	CGC	85.81 ± 3.8%
	VC671	*egl-3 ( ok979 ) V. *	CGC	68.11 ± 1.6%
	CHS10032	*flp-1 (yum393) IV; flp-23 (yum394) III; flp-14 (yum423) III; flp-25 (yum424) III. *	This study	10.00 ± 1.9%
	CHS10149	*nlp-12 (yum458) I; nlp-39 (yum500) I; capa-1 (yum522) X; nlp-6 (yum537) X. *	This study	8.89 ±1.1%
	CHS10088	*nlp-60 (yum462) IV; nlp-68 (yum463) III; nlp-67 (yum538) X. *	This study	8.89 ±1.1%
	CHS10025	*ins-26 (yum386) I; ins-32 (yum387) II; ins-9 (yum486) X; ins-13 (yum487) II. *	This study	7.78 ± 2.2%
	CHS10183	*nlp-30 (yum420) V; nlp-28 (yum548) V; nlp-29 (yum549) V; nlp-31 (yum550) V. *	This study	7.78 ± 1.1%
	CHS10011	*nlp-19 (yum425) X; nlp-62 (yum426) I; ntc-1 (yum478) X; nlp-64 (yum479) X. *	This study	6.67 ± 1.9%
	CHS10110	*nlp-41 (yum501) II; nlp-45 (yum502) X; nlp-17 (yum528) IV; nlp-77 (yum529) II. *	This study	6.67 ± 3.3%
	CHS10057	*ins-25 (yum388) I; ins-28 (yum389) I; ins-5 (yum409) II; ins-29 (yum410) I; ins-27 (yum455) I. *	This study	5.56 ± 2.9%
	CHS10009	*flp-12 (yum400) X; flp-21 (yum401) V; flp-5 (yum422) X; flp-24 (yum423) III; flp-28 (yum475) X. *	This study	5.56 ± 2.2%
	CHS10091	*nlp-48 (yum467) III; nlp-52 (yum468) III; nlp-40 (yum541) I; nlp-78 (yum542) II. *	This study	5.56 ± 1.1%
	CHS10073	*ins-2 (yum391) II; ins-34 (yum392) IV; ins-12 (yum428) II; ins-15 (yum429) II; ins-11 (yum508) II. *	This study	4.44 ± 2.9%
	CHS10010	*ins-10 (yum413) V; ins-19 (yum414) II; ins-35 (yum476) V; ins-36 (yum477) I. *	This study	3.33 ± 1.9%
	CHS10118	*ins-20 (yum382) II; ins-30 (yum383) I; daf-28 (yum514) V; ins-24 (yum515) I; ins-33 (yum534) I. *	This study	3.33 ± 1.9%
	CHS10065	*nlp-1 (yum551) X; nlp-38 (yum552) I; nlp-3 (yum553) X; nlp-13 (yum554) V. *	This study	3.33 ± 1.9%
	CHS10093	*nlp-34 (yum419) nlp-33 (yum503) nlp-27 (yum504) nlp-25 (yum520) V. *	This study	3.33 ± 1.9%
	CHS10095	*nlp-4 9(yum417) X; nlp-51 (yum418) II; nlp-22 (yum461) X; nlp-46 (yum546) V; nlp-71 (yum547) IV. *	This study	3.33 ± 1.9%
	CHS10085	*flp-10 (yum472) IV; flp-27 (yum473) II; flp-34 (yum395) V; flp-11 (yum509) X. *	This study	2.22 ± 1.1%
	CHS10006	*flp-7 (yum436) X; flp-9 (yum437) IV; flp-17 (yum405) IV; flp-22 (yum406) I. *	This study	2.22 ± 1.1%
	CHS10148	*nlp-66 (yum427) X; nlp-11 (yum480) II; nlp-5 4(yum535) IV; nlp-79 (yum536) IV. *	This study	2.22 ± 1.1%
	CHS10013	*nlp-16 (yum432) IV; nlp-55 (yum433) II; nlp-8 (yum481) I; nlp-61 (yum482) X. *	This study	2.22 ± 1.1%
	CHS10092	*nlp-23 (yum469) X; nlp-59 (yum470) V; nlp-35 (yum523) IV; pdf-2 (yum524) X. *	This study	2.22 ± 1.1%
	CHS10102	*nlp-9 (yum415) V; nlp-32 (yum416) III; nlp-26 (yum460) V; nlp-24 (yum543) V. *	This study	2.22 ± 1.1%
	CHS10111	*nlp-56 (yum459) IV; nlp-57 (yum543) X; nlp-63 (yum544) II; nlp-53 (yum545) X. *	This study	2.22 ± 1.1%
	CHS10034	*flp-3 (yum396) X; flp-6 (yum397) V; flp-13 (yum430) IV; flp-18 (yum431) X. *	This study	1.11 ± 1.1%
	CHS10040	*nlp-4 (yum451) I; nlp-80 (yum452) V; nlp-18 (yum489) II; nlp-42 (yum490) V. *	This study	1.11 ± 1.1%
	CHS10089	*nlp-70 (yum464) V; nlp-76 (yum510) X; nlp-36 (yum511) III. *	This study	1.11 ± 1.1%
	CHS10109	*nlp-21 (yum445) III; nlp-69 (yum446) V; nlp-73 (yum505) V; pdf-1 (yum527) III. *	This study	1.11 ± 1.1%
	CHS10046	*ins-3 (yum384) II; ins-21 (yum385) III; ins-22 (yum407) III; ins-23 (yum456) III. *	This study	1.11 ± 1.1%
	CHS10062	*ins-17 (yum493) III; ins-18 (yum494) I; ins-16 (yum495) III; ins-37 (yum496) II. *	This study	0.00 ± 0%
	CHS10081	*ins-14 (yum402) II; ins-31 (yum408) II; ins-39 (yum444) X; ins-1 (yum497) IV. *	This study	0.00 ± 0%
	CHS10080	*ins-4 (yum390) II; ins-6 (yum411) II; ins-7 (yum412) IV; ins-8 (yum521) IV. *	This study	0.00 ± 0%
	CHS10033	*flp-4 (yum398) II; flp-15 (yum399) III; flp-2 (yum421) X; flp-16 (yum422) II. *	This study	0.00 ± 0%
	CHS10063	*flp-20 (yum403) X; flp-32 (yum404) X; flp-19 (yum457) X; flp-33 (yum498) I; flp-26 (yum499) X. *	This study	0.00 ± 0%
	CHS10084	*nlp-20 (yum440) IV; nlp-43 (yum441) III; msrp-7 (yum506) II; lury-1 (yum507) III. *	This study	0.00 ± 0%
	CHS10103	*nlp-5 (yum449) II; nlp-10 (yum450) III; nlp-2 (yum488) X; nlp-50 (yum531) II. *	This study	0.00 ± 0%
	CHS10119	*nlp-58 (yum465) V; nlp-15 (yum512) I; nlp-14 (yum513) X; nlp-47 (yum539) IV. *	This study	0.00 ± 0%
**Others**	KP1097	*dgk-1 ( nu62 ) X. *	CGC	10.00 ± 8.3%
	MT1083	*egl-8 ( n488 ) V. *	CGC	7.78 ± 4.4%
	DG1856	*goa-1 ( sa734 ) I. *	CGC	6.67 ± 1.9%
	MT6308	*eat-4 ( ky5 ) III. *	CGC	5.56 ± 1.1%
	KP4	*glr-1 ( n2461 ) III. *	CGC	4.44 ± 1.1%
	NL2105	*gpa-3 ( pk35 ) odr-3 ( n1605 ) V. *	CGC	4.10 ± 1.3%
	CX7102	*qaIs2241 [ gcy-36 :: egl-1 ; gcy-35 ::GFP; lin-15 (+)] X. *	CGC	3.33 ± 0.9%
	VC2393	*acy-2 ( ok3003 ) V. *	CGC	3.33 ± 1.9%
	BS3383	*pmk-3 ( ok169 ) IV. *	CGC	2.22 ± 1.1%
	RB754	*aak-2 ( ok524 ) X. *	CGC	2.22 ± 2.2%
	RB1231	*pde-4 ( ok1290 ) II. *	CGC	2.22 ± 2.2%
	JT307	*egl-9 ( sa307 ) V. *	CGC	1.11 ± 1.1%
	CB1370	*daf-2 ( e1370 ) III. *	CGC	1.11 ± 1.1%
	RB2302	*daf-7 ( ok3125 ) III. *	CGC	1.11 ± 1.1%
	DA609	*npr-1 ( ad609 ) X. *	CGC	1.11 ± 1.1%
	RB1920	*mig-10 ( ok2499 ) III. *	CGC	1.11 ± 1.1%
	BX24	*fat-1 ( wa9 ) IV. *	CGC	1.11 ± 1.1%
	IB16	*ceh-17 ( np1 ) I. *	CGC	0.00 ± 0%
	GJ7	*gpa-2 ( pk16 ) gpa-3 ( pk35 ) gpa-13 ( pk1270 ) V; gpa-5 ( pk376 ) gpa-6 ( pk480 ) X. *	CGC	0.00 ± 0%
	MT1073	*egl-4 ( n478 ) IV. *	CGC	0.00 ± 0%
	STE68	*nhr-49 ( nr2041 ) I. *	CGC	0.00 ± 0%
	IK130	*pkc-1 ( nj3 ) V. *	CGC	0.00 ± 0%
	PS3551	*hsf-1 ( sy441 ) I. *	CGC	0.00 ± 0%
	RB674	*stam-1 ( ok406 ) I. *	CGC	0.00 ± 0%
	RB711	*pqm-1 ( ok485 ) II. *	CGC	0.00 ± 0%
	JT1058	*hid-1 ( sa1058 ) X. *	CGC	0.00 ± 0%
	BX106	*fat-6 ( tm331 ) IV. *	CGC	0.00 ± 0%
	BX30	*fat-3 ( wa22 ) IV. *	CGC	0.00 ± 0%
	KP1182	*acy-1 ( nu329 ) III. *	CGC	0.00 ± 0%
	BX26	*fat-2 ( wa17 ) IV. *	CGC	0.00 ± 0%
	BX17	*fat-4 ( wa14 ) IV. *	CGC	0.00 ± 0%
	ZG31	*hif-1 ( ia4 ) V. *	CGC	0.00 ± 0%
	GR2245	*skn-1 ( mg570 ) IV. *	CGC	0.00 ± 0%

## Extended Data

Description: A short video clip showing how animals react when they encounter the bacteria in 1% O2. Resource Type: Audiovisual. DOI: 10.22002/aknxd-92g65

## References

[R1] Abergel Z, Shaked M, Shukla V, Wu ZX, Gross E (2021). The phosphatidylinositol transfer protein PITP-1 facilitates fast recovery of eating behavior after hypoxia in the nematode Caenorhabditis elegans.. FASEB J.

[R2] Brenner S (1974). The genetics of Caenorhabditis elegans.. Genetics.

[R3] Busch KE, Laurent P, Soltesz Z, Murphy RJ, Faivre O, Hedwig B, Thomas M, Smith HL, de Bono M (2012). Tonic signaling from O₂ sensors sets neural circuit activity and behavioral state.. Nat Neurosci.

[R4] Cheung BH, Cohen M, Rogers C, Albayram O, de Bono M (2005). Experience-dependent modulation of C. elegans behavior by ambient oxygen.. Curr Biol.

[R5] Couto A, Oda S, Nikolaev VO, Soltesz Z, de Bono M (2013). In vivo genetic dissection of O2-evoked cGMP dynamics in a Caenorhabditis elegans gas sensor.. Proc Natl Acad Sci U S A.

[R6] Dokshin Gregoriy A, Ghanta Krishna S, Piscopo Katherine M, Mello Craig C (2018). Robust Genome Editing with Short Single-Stranded and Long, Partially Single-Stranded DNA Donors in
*Caenorhabditis elegans*. Genetics.

[R7] Ghanta Krishna S, Mello Craig C (2020). Melting dsDNA Donor Molecules Greatly Improves Precision Genome Editing in
*Caenorhabditis elegans*. Genetics.

[R8] Gray JM, Karow DS, Lu H, Chang AJ, Chang JS, Ellis RE, Marletta MA, Bargmann CI (2004). Oxygen sensation and social feeding mediated by a C. elegans guanylate cyclase homologue.. Nature.

[R9] Gross E, Soltesz Z, Oda S, Zelmanovich V, Abergel Z, de Bono M (2014). GLOBIN-5-dependent O2 responses are regulated by PDL-1/PrBP that targets prenylated soluble guanylate cyclases to dendritic endings.. J Neurosci.

[R10] Jacob TC, Kaplan JM (2003). The EGL-21 carboxypeptidase E facilitates acetylcholine release at Caenorhabditis elegans neuromuscular junctions.. J Neurosci.

[R11] Kaelin WG Jr, Ratcliffe PJ (2008). Oxygen sensing by metazoans: the central role of the HIF hydroxylase pathway.. Mol Cell.

[R12] Kass J, Jacob TC, Kim P, Kaplan JM (2001). The EGL-3 proprotein convertase regulates mechanosensory responses of Caenorhabditis elegans.. J Neurosci.

[R13] Kayser B, Verges S (2021). Hypoxia, energy balance, and obesity: An update.. Obes Rev.

[R14] Kietzmann T, Mäkelä VH (2021). The hypoxia response and nutritional peptides.. Peptides.

[R15] Laurent P, Soltesz Z, Nelson GM, Chen C, Arellano-Carbajal F, Levy E, de Bono M (2015). Decoding a neural circuit controlling global animal state in C. elegans.. Elife.

[R16] Onukwufor JO, Farooqi MA, Vodičková A, Koren SA, Baldzizhar A, Berry BJ, Beutner G, Porter GA Jr, Belousov V, Grossfield A, Wojtovich AP (2022). A reversible mitochondrial complex I thiol switch mediates hypoxic avoidance behavior in C. elegans.. Nat Commun.

[R17] Quintero P, Milagro FI, Campión J, Martínez JA (2009). Impact of oxygen availability on body weight management.. Med Hypotheses.

[R18] Semenza GL (2009). Defining the role of hypoxia-inducible factor 1 in cancer biology and therapeutics.. Oncogene.

[R19] Van Voorhies WA, Ward S (2000). Broad oxygen tolerance in the nematode Caenorhabditis elegans.. J Exp Biol.

[R20] Westerterp KR, Kayser B, Wouters L, Le Trong JL, Richalet JP (1994). Energy balance at high altitude of 6,542 m.. J Appl Physiol (1985).

[R21] Zhao L, Fenk LA, Nilsson L, Amin-Wetzel NP, Ramirez-Suarez NJ, de Bono M, Chen C (2022). ROS and cGMP signaling modulate persistent escape from hypoxia in Caenorhabditis elegans.. PLoS Biol.

[R22] Zimmer M, Gray JM, Pokala N, Chang AJ, Karow DS, Marletta MA, Hudson ML, Morton DB, Chronis N, Bargmann CI (2009). Neurons detect increases and decreases in oxygen levels using distinct guanylate cyclases.. Neuron.

